# With a little help from my friends? Perceived friendship quality and narrative identity in adolescence

**DOI:** 10.1111/jora.12605

**Published:** 2021-02-23

**Authors:** Elisabeth L. de Moor, Jolien Van der Graaff, Lotte van Doeselaar, Theo A. Klimstra, Susan Branje

**Affiliations:** ^1^ Department of Youth and Family Utrecht University Utrecht the Netherlands; ^2^ Department of Developmental Psychology Tilburg University Tilburg the Netherlands; ^3^ Department of Child Study and Human Development Tufts University Medford MA USA

**Keywords:** adolescence, perceived friendship quality, redemption, self‐event connections, turning point narratives

## Abstract

Stressful events are associated with various outcomes, but there is variability in these associations suggesting that the interpretation of these events is important. This interpretation is reflected in the narratives adolescents tell of events, which are largely constructed in social interactions. We examined the associations of perceived friendship quality with self‐event connections and redemption in turning point narratives, in a sample of Dutch adolescents. Findings from regression analyses in a cross‐sectional subsample (*N* = 1087, *M*_age_ = 14.8) and a three‐wave cross‐lagged panel model in a longitudinal subsample (*N* = 186, *M*_age at Wave 1_ = 14.7) showed that perceived friendship quality was associated with the presence of redemption sequences and self‐event connections within time points, but not longitudinally.

## INTRODUCTION

Finding a sense of personal identity is considered a key developmental task in adolescence (Erikson, 1950, 1968). From early adolescence onward, young people are expected to start creating a meaningful and coherent account of who they are, how they came to be that person, and who they might wish to become in the future (McAdams, 1985). This process concerns making sense of one’s own role, not only in daily experiences and interactions but also in major events (McLean & Jennings, 2012; Pasupathi & Weeks, 2010). Moreover, adolescents need to create a sense of personal continuity across time and situations and maintain that continuity by making sense of their experiences and integrating them into their life story (e.g., McAdams, 1993, 2001a; Pasupathi & Mansour, 2006).

Making sense of the events one experiences is an important aspect of identity development (Graber et al., 1996). Research has often evidenced large variability in the link between events and adjustment (e.g., Grant et al., 2006; De Moor et al., 2019). For instance, whereas stressful events have been found to predict mental health problems for some adolescents (Laceulle et al., 2014), they are associated with post‐event growth in life satisfaction in others (Meyerson et al., 2011), and for again others there may be no link with adjustment at all. This suggests that the meaning that adolescents ascribe to events is more important for understanding post‐event adjustment than the experience of the event itself (e.g., Skaggs & Barron, 2006). How people deal with significant events in their lives is reflected in their autobiographical narratives of these events. These narratives have been shown for a large part to be constructed in social interactions (Fivush et al., 2011). Therefore, it is possible that differences in relationship quality with important others may explain differences in the way adolescents are able to construct narratives to deal with and interpret events in light of their identity.

In the present study, we examined whether perceived friendship quality was related to the narratives adolescents construct about an event that they consider a turning point in their lives. In these narratives, we focused specifically on the integration of the event into the life narrative, and the ways in which the event was interpreted. We investigated longitudinally whether perceived friendship quality predicted these narrative identity processes, and vice versa, whether the quality of the narratives predicted higher perceived friendship quality at a later time point.

### Narrative identity: Self‐event connections and redemption sequences

Life stories start to become internalized in adolescence (Habermas & Paha, 2001; McAdams, 1985; McAdams & McLean, 2013; McLean et al., 2010), and as such, we may expect that the aspects of these narratives that represent more developed identity processes become more pronounced across this life stage. In the present study, we focused on the role of two aspects of narratives that have been found to be important for understanding identity formation and well‐being (Adler et al., 2016; McLean et al., 2019): self‐event connections and redemption sequences, during the period of middle adolescence.

In order to create a coherent account of who they are as a person, adolescents need to identify temporal consistencies in their life narrative, as well as explain inconsistencies. Within the context of a singular event, this process is termed the making of self‐event connections (Pasupathi et al., 2007). Self‐event connections are explicit connections that individuals make between events and their self‐concept. By making self‐event connections, individuals specify what an event says about them as a person. For instance, adolescents may use events to explain a consistency in their person (e.g., “I always put off studying until the last moment and that’s why I failed the test”), or to explain any inconsistencies (e.g., “I always thought I was conscientious, but failing this test caused me to see that I wasn’t as good at preparing for an exam as I thought I was. That made me become more conscientious about my schoolwork”). Within the life narrative, the making of self‐event connections allows adolescents to add new aspects to the story or to change existing ones. As such, self‐event connections may serve as the mechanism through which individuals form their identity, as well as keep their narrative identity “up‐to‐date.” The making of self‐event connections is considered vital for the development and maintenance of identity, and has empirically been linked to higher psychological well‐being (Bauer & McAdams, 2004) and a more committed identity (Van Doeselaar et al., 2019; McLean & Pratt, 2006).

Every event may signify a point of consistency or inconsistency. Therefore, each event has the potential for both change and stability connections. However, it is only when adolescents see events as important for who they are, that they will incorporate them into their life narrative and that identity change or stabilization will occur (Dunbar & Grotevant, 2004; McLean et al., 2010). In general, adolescents have been found to be more likely to make connections that explain inconsistencies (i.e., change) than to make connections that explain consistencies (i.e., stability; McLean, 2008). This is even more the case for turning point narratives, as these narratives describe a point in adolescents’ lives at which they experienced a change in themselves or in their understanding of themselves (e.g., Pasupathi et al., 2007). While biased toward events that explain inconsistencies, turning point narratives provide key insights into the events that adolescents perceive as having contributed to who they presently are. As such, turning point narratives may be especially informative for examining self‐event connections.

In addition to deciding on whether or not an event is important for their narrative identity, adolescents ascribe general meaning to events. While events often are objectively positive or negative in valence, more important is the subjective positivity versus negativity that individuals ascribe to the aftermath of these events. That is, an event may be considered negative at the time (e.g., having to repeat a grade) but may in the long run come to represent a positive change in adolescents’ lives (e.g., meeting their best friend). This process is termed redemption (McAdams et al., 1997). While some events may have more or less objectively positive valence, whether these events are integrated in the life story as positive or negative moments in their lives depends largely on how adolescents interpret them. Whether the event becomes regarded as a positive or negative event may depend on adolescents’ desire and ability to actively link together scenes in their life story, to regain a sense of progress and optimism.

The presence of redemption sequences in narratives has been linked to self‐esteem in adolescent boys (McLean & Breen, 2009). Moreover, individuals who use redemption in their life narrative generally report higher well‐being (McAdams et al., 2001), possibly because they are more optimistic about life in general and consequently about events in their own lives. Making redemption sequences might also cause individuals to become happier, as they see something positive in a previously negatively regarded event.

### Individual differences in narrative identity

Whether or not adolescents engage in these narrative identity processes is dependent on both individual and contextual circumstances, and in general some adolescents are more likely to engage in narrative identity processes than others (McAdams, 2001b; McLean et al., 2010). In part, the characteristics of adolescents’ narrative identities have been found to depend on age (e.g., Habermas & Paha, 2001; Köber et al., 2015; Reese et al., 2014), culture (Leichtman et al., 2003; McAdams, 2004), and gender (McLean, 2008).

As narratives are for a large part constructed in a social context, the social context may also be involved in making some adolescents more likely to deduce meaning—and deduce certain kinds of meaning—from their experiences than others. In line with this, previous research has shown that experiencing good relationships with peers is an important factor in the development of adolescents’ personal identities (Meeus et al., 2002; De Moor et al., 2019). The social context may be involved in predicting these individual differences in two different ways: by processes that help adolescents develop their narrative identity within the social context and by the way the social context contributes to narrative identity development in other areas of adolescents’ lives.

In early childhood, processes within social relationships already start to play an important role in narrative development, as parents scaffold the development of their children’s early storytelling skills that form the foundation for the development of their narrative identity (Fivush et al., 2006). They do this by providing structure and support, and by asking for elaboration on particular aspects of children’s narrating (e.g., “how did that event make you feel?”; Fivush et al., 2006). Responsiveness to the stories youth tell—and the way they tell those stories—remains of importance in adolescence. However, during this period peers start to play a more prominent role in providing companionship and support (Scholte et al., 2001) and become the most common conversation partner for adolescents (Raffaelli & Duckett, 1989). Indeed, research that asked adolescents to narrate about a critical personal event to their mother and a friend showed that scaffolding behavior by both types of conversation partners stimulates adolescents to derive meaning from their experiences, and that particularly friends might provide a safe arena to try out newly constructed narratives (McLean & Jennings, 2012). Moreover, adolescents who had conversations with friends who were more responsive were more likely to interpret and derive meaning from everyday experiences (Pasupathi & Hoyt, 2009). Friends could thus play an important role in adolescent narrative identity development.

Not only the listening behavior of the friend but also the topics that are discussed may contribute to the development of narrative identity. Self‐disclosure and intimacy are prominent in the conversations of adolescents and their friends (e.g., Berndt, 2002; Vijayakumar & Pfeifer, 2020), and this is particularly true for high‐quality friendships. Self‐disclosure and intimacy are considered to play an important role in adolescent self‐exploration and self‐understanding (Gottman & Mettetal, 1986). As such, high‐quality friendships are thought to be related to better outcomes because they provide a climate in which adolescents can discuss important events and in which they feel supported in thinking more deeply about the meaning of these experiences. This may, in turn, result in the making of links between the self and events, and could facilitate positive reinterpretation of negative events.

Moreover, high‐quality friendships may also help adolescents to develop their identity outside of the immediate social context. High‐quality friendships are characterized by a mutual feeling of love, by caring for and comforting each other, and by providing a safe basis for exploration in various domains of life (e.g., Ainsworth, 1989; Bowlby, 1969; Furman & Buhrmester, 2009). They facilitate exploration by providing adolescents with the social resources to cope with setbacks and provide a safe place from where to move forward. Thus, perceived friendship quality may contribute to the development of adolescents’ narrative identity in a direct and a more indirect manner.

### Narrative Identity as a Predictor of Friendship Quality

Individual differences in the making of self‐event connections and redemption sequences may not only be explained by perceived friendship quality, but may also contribute to the development of these friendships. Following Erikson’s model of key developmental tasks (1950), a sense of trust promotes identity formation, and developing a clear sense of identity is pivotal for having meaningful relationships with others (i.e., achieving intimacy). Individuals with a more developed identity are thought to be better able to convey their identity to their conversation partner, thus allowing them more time and headspace to manage actual goals of the interaction such as providing or seeking support (Swann, 2000; Swann, Milton, & Polzer, 2000). In addition to differences on the side of the adolescent, being aware of who one is, contributes to the ease with which adolescents can be read by others (i.e., judgeability; Colvin, 1993; Human & Biesanz, 2013). This may in turn contribute to the quality of friendships by increasing familiarity and social support in the relationship. In line with this, previous research on identity found that adolescents with a clearer self‐concept, who had a clearer and more consistent view of who they are, reported feeling more supported in the relationships with their friends one year later (Becht et al., 2017). As such, adolescents with a more developed identity could be expected to experience higher friendship quality.

Furthermore, especially the combination of making self‐event connections and redemption sequences may be important for perceived friendship quality. Narratives in which adolescents make connections between an event and their self *and* in which they see that an initially negative event has a positive influence on their life, may be the most impactful for their self‐understanding. That is, finding meaning in experiences may be especially important when these experiences are regarded as positive for one’s life narrative, thus signifying greater growth in identity. This greater sense of self may, in turn, affect adolescents’ friendships. However, empirical work has yet to examine whether narrative identity, and specifically the interaction between several kinds of narrative identity processes, is predictive of changes in adolescents’ perceptions of their friendship quality.

### Current study

While previous research has shown the importance of linking events to the self and being able to redeem negative events for identity development and for general well‐being (McAdams et al., 2001; Pasupathi et al., 2007), few studies have examined what factors in adolescents’ environments may explain individual differences in the extent to which adolescents engage in narrative identity processes. Identity formation is for a large part thought to occur in interaction with others (Pasupathi & Weeks, 2010) and scaffolding by friends was found to be related to a more developed narrative identity in a conversational storytelling context (McLean & Jennings, 2012). As such, the present study examined the links of perceived friendship quality with self‐event connections and redemption sequences.

In the first part of the study, we examined the links between perceived friendship quality, self‐event connections, and redemption sequences cross‐sectionally, where it was expected that perceived friendship quality would be positively related to the narrative identity processes of making self‐event connections and redemption sequences. Moreover, as narrative identity in general becomes more developed across adolescence, we expected a positive link between making self‐event connections and redemption sequences in a narrative. In the second part of the study, we examined these associations as they developed across three annual time points. Bidirectional links were hypothesized, with higher perceived friendship quality predicting narrative processes (i.e., both the making of connections between an event and the self and the making of redemption sequences), and these narrative processes in turn predicting higher friendship quality. In addition, we examined whether the interaction of making self‐event connections and redemption was predictive of perceived friendship quality. It was expected that especially when adolescents made redemption sequences for that event, the making of self‐event connection would be predictive of higher friendship quality. Our research questions and hypotheses, methods, and analysis plan were preregistered at https://osf.io/fvbjs. Deviations from our original plan are described in Table S1 in the Online Supplementary Material.

## METHOD

### Sample and procedure

To examine the bidirectional links of self‐event connections and redemption sequences with perceived friendship quality, we used data from the first three annual waves of the ongoing Project‐Me study (total *N* = 1941). For the cross‐sectional analyses, we included adolescents who both wrote a turning point narrative and had friendship quality data at Wave 1 (*n* = 1087). The cross‐sectional sample had a mean age of 14.8 years (*SD* = 0.74), and approximately half of the participants were female (55.7%). Of the 1087 adolescents who participated in our cross‐sectional sample, 147 participated in the longitudinal part of Project‐Me and on at least one more time point wrote a turning point narrative in addition to providing data on friendship quality. In addition, 41 adolescents who did not provide both a narrative and friendship quality data at Wave 1, but did participate at Wave 2 and Wave 3 were included in the longitudinal sample (*n* = 186, 9.6% of the total cross‐sectional sample). Of this sample, mean age of the adolescents at Wave 1 was 14.7 years (*SD* = 0.69; Wave 2: *M* = 15.9, *SD* = 0.76, Wave 3: *M* = 16.8, *SD* = 0.66) and 62.4% was female. The participants came from all three academic tracks in the Dutch secondary school system which, based on the educational level, we have termed low, middle, and high (see Table 1). Information about the ethnic background of our participants was not available in Wave 1, but was for the succeeding waves. For our longitudinal sample, the distribution indicates that most adolescents identified as Dutch (95.2%) with only a small subgroup using a different, self‐chosen ethnic label (4.8%; see Table 1). Of these, Moroccan and African were the most common and the only ones chosen by more than 1% of the sample (both 1.1%).

**Table 1:** Descriptive statistics of the sample

	Cross‐sectional (*n* = 1,087)					Longitudinal (*n* = 186)			
	Wave 1			Wave 1		Wave 2		Wave 3	
	*M* (*SD*)/*N* (%)		Range	*M* (*SD*)/*N* (%)	Range	*M* (*SD*)/*N* (%)	Range	*M* (*SD*)/*N* (%)	Range
Age	14.82 (0.74)		12.74–17.44	14.73 (0.69)	13.00–16.69	15.89 (0.76)	13.93–17.85	16.78 (0.66)	15.13–18.67
Gender (female)	605 (55.7%)			116 (62.4%)					
Educational level at Wave 1									
Low	189 (17.4%)			13 (7.0%)					
Middle	400 (36.8%)			47 (25.3%)					
High	498 (45.8%)			126 (67.7%)					
Ethnic background									
Dutch				177 (95.2%)					
Other				9 (4.8%)					
Self‐event connections (yes)	509 (46.8%)			102 (56.7%)		95 (69.9%)		78 (60.9%)	
Redemption (yes)	205 (18.9%)			48 (26.7%)		26 (19.1%)		33 (25.8%)	
Friendship quality		3.03 (1.04)	1–5	3.08 (0.97)	1–5	2.94 (0.95)	1–5	3.12 (0.97)	1–5

We approached various high schools in the south of The Netherlands and seven of these decided to collaborate. In the cross‐sectional part of the study, adolescents had to provide active assent with their participation (or consent when they were 16 years old or older). Parents provided passive consent, meaning that they were handed a letter and were given two weeks to return the letter in case they did not want their child to participate in the study. Out of the 2130 adolescents who were approached at the seven participating schools, we obtained consent for 91.2% of the adolescents and their parents. Subsequently, adolescents independently filled out questionnaires and wrote their turning point narratives on computers in classrooms during one class hour (45 or 50 min). Graduate students were present to guide the process and provide instructions. Adolescents did not receive incentives for their participation at Wave 1.

One year later, adolescents and their caregivers were informed about the longitudinal part and were requested to provide assent and consent for adolescents’ participation, respectively. Again, adolescents who were 16 years or older provided consent rather than assent. Adolescents who had not responded at Wave 2 were again contacted a year later at Wave 3. This time caregivers were asked to provide passive consent when adolescents were 16 or older and active consent when adolescents were younger than 16 years old. Adolescents completed the questionnaires (including turning point narratives) in their own time. They received 5 euros for participation at Wave 2 and 10 euros at Wave 3. The cross‐sectional and longitudinal parts of the study were both approved by the local institutional review board (IRB; protocol number EC‐2015.49).

### Measures

#### Perceived friendship quality

We measured perceived friendship quality with 12 items from the Network of Relationship Inventory—Behavioral System Version (NRI‐BSV; Van Aken & Hessels, 2012; Furman & Buhrmester, 2009) measuring adolescents’ perceptions of received and provided friendship support at all three waves. Adolescents were asked to report on the quality of the relationship with their best friend. For our measure of friendship quality, we selected all positive relationship quality scales (i.e., “provides safe haven,” “provides secure base,” “seeks safe haven,” and “seeks secure base”) with the exception of the “companionship” subscale, as it pertains to time spent together rather than the quality of this time. A complete overview of the items of the scales that we used is provided in Table S5 in the Online Supplementary Material. The items were rated on a 5‐point Likert scale, ranging from 1 (*little or none*) to 5 (*almost always*). Past work has found acceptable validity and reliability of the entire scale (e.g., Edens et al., 1999), as well as adequate factor loadings of the subscales on the support factor (Furman & Buhrmester, 2009). Internal consistency as estimated with coefficient alpha ranged from .94 and .95 across the three waves of the present study.

#### Self‐event connections

We measured self‐event connections by asking adolescents to write a turning point narrative at all three waves (for the English prompt, see McLean et al., 2010, as adapted from McAdams, 2008). Participants were asked to write about an event in their life which they viewed as a turning point in their self‐understanding. They were asked to write what had happened, when it had happened and who was involved, and what they were thinking and feeling at the time of the event. In addition, they were asked to describe why the event was significant and what it may say about them and their personality.

The resulting narratives were coded to capture whether adolescents made one (or more) of four different types of connections between the event and the self, based on a coding system developed by Pasupathi et al. (2007; and adapted by Lilgendahl & McLean, 2020). Following this system, we coded for the presence of a self‐event connection when adolescents made explicit statements concerning the link between the self and the event. They could do this by means of illustration/explanation (“The event is explained by or illustrates some trait or quality that I possess”), dismissal (“I give a self‐description to make sure that you, the audience, don’t develop a particular opinion about me”), causation (“The event made me a certain type of person, provided me with a certain skill, or induced a certain goal”), or revelation (“The event revealed that I’m a person who is …”). Any of these links were coded as the presence of a self‐event connection. For a self‐event connection to be coded as present, the connection had to be explicit, in the sense that the exact spot could be marked in the text. Only connections between events and the current self were coded; connections with an aspect of the self that had changed again since the event were not coded (e.g., “This event made me more conscientious about school for some time, but I have now gone back to being more laid back about it”). This was done because an event that has only links with a past self is not connected to the narrative about the current self and no longer provides a sense of coherence and continuity. The narratives were coded by a group of graduate and undergraduate coders. Before coding commenced, coders were trained in two steps. First, a group of researchers including the graduate coders discussed the existing coding system with a subsample of narratives (10% of all narratives at Wave 1), and adapted the coding system to the present sample where necessary. Using the adapted system, codes were determined for the subsample of narratives. Second, undergraduate coders were trained using the already coded narratives. After completing training, all narratives were coded by three (Wave 1) or two (Wave 2 and 3) coders. This means that each narrative was coded by two or more independent coders, but that subsamples of narratives were coded by different groups of coders. In case of disagreement, the coders discussed their codes until consensus was reached. All coders coded approximately the same amount of narratives. Reliability (κ) of the initial coding (i.e., before discussion to reach consensus) of all narratives across the coders was .72 in the cross‐sectional sample (86% intercoder agreement) and .78 in the longitudinal sample (89% agreement). For the present study, we examined per narrative if one or more self‐event connections were made, which was then dichotomized into a score of 0 (no connection) or 1 (one or more connections).

#### Redemption

Themes of redemption in turning point narratives were coded independently from self‐event connections based on the coding system developed by McAdams (1999). In this coding system, redemption was coded when a narrative showed clear and explicit signs of an objectively negative‐affect state or an affect state that was experienced as negative by the participant, which later turned into a decidedly positive‐affect state. In Project‐Me, presence of a redemption sequence was coded using a scale from 0 to 2. A score of 0 was given when there was no final positive‐affect state in the narrative, when there was no initial negative state, or when the valence of states was ambiguous. Narratives received a 1 when they ended with a positive‐affect state and started with a negative state, without the positive end state being connected to a reinterpretation of the negative event. In the case that these positive‐affect states followed a negative‐affect state and the positive states were based on a reinterpretation of the initially negative event, stories received a 2. In the present study, we defined redemption similarly as in previous research, as a narrative with an initial negative‐affect state and a positive end state (e.g., McLean & Breen, 2009). Thus, in line with past work (e.g., Alea, 2018; McAdams et al., 2001; McCoy & Dunlop, 2017), we dichotomized the initial scores into 0 (*no redemption; 0 in our original coding*) and 1 (*redemption; a 1 or 2 in our original coding*). Redemption was coded by the principal investigator of the project together with a small group of graduate students. Like for self‐event connections, training of the coders was conducted in two steps, after which all narratives were coded on redemption sequences independently by three coders. Reliability (κ) of the initial redemption coding across all three coders was calculated for all coded narratives, and was .63 for the cross‐sectional data (84% intercoder agreement) and .66 for the longitudinal data (85% agreement). Cases of disagreement were discussed until consensus was reached. Table 2 provides sample narratives that are composed of several narratives from participants and their coding on self‐event connections and redemption.

**Table 2:** Samples of turning point narratives and their coding on self‐event connections and redemption

	Coding scheme		
Turning point narrative	Self‐event connections (0 or 1)	Redemption (0 or 1)	Redemption alternative (0 or 1)
“I often keep to myself and keep my contact with other classmates to a minimum. I was walking home from school one day, I was sure I saw a man cycling towards me. As I got closer, I realized that the man disappeared. I thought it was strange. Since then I often feel that someone is around, even though I mostly go everywhere alone.”	0	0	0
“My parents were divorced and years later my father got a new girlfriend in another city and we had to move there. I was excited about it at first, but when it got closer to the move, it got harder and harder to leave. When I finally moved, I went through a hard time and I still find it equally difficult. I got a pet cat later on, and felt less lonely and better.”	0	1	0
“My grandmother passed away from cancer. I heard it when I came home from school. I felt sad because my grandmother and I were really close. It changed me in the sense that I know that everyone will die at some point and that I also should expect this a little. Since then I know that I want to be an oncologist.”	1	1	1
“I was told that I had to go to a class of a lower level, because my grades were bad. I was very annoyed by this. It made me realize that school has a big influence on what I can do with my life in the future. I became more serious at school and noticed that my grades had improved.”	1	1	1

Note

The table and its contents were adapted from a recent paper on the same dataset by See et al. (in press). The examples presented here are composed of narratives by two or three adolescents about a similar event. Changes were also made to ensure anonymity. In the present study, redemption was defined as a narrative in which there is an initial negative‐affect state and a positive‐affect end state. In the alternative coding of redemption, redemption was only coded as present when the change from negative to positive affect was accompanied by an explicit reinterpretation by the adolescent.

### Data analytical plan

#### Attrition analyses

To examine attrition in our samples, we first conducted Little’s (1988) missing completely at random (MCAR) test in IBM SPSS version 25.0. We found that missingness on perceived friendship quality and the narrative identity characteristics across the waves was not at random for the complete Project‐Me sample, *χ* ^2^(141) = 175.05, *p* = .027. For the cross‐sectional sample in the present study, for which we only included participants who wrote a turning point narrative and had data on perceived friendship quality at Wave 1, missingness was found to be random, *χ* ^2^(55) = 62.92, *p* = .216. In comparison to adolescents who were not selected for this study, participants in our cross‐sectional sample were relatively older, *t*(1754.40) = −6.76, *p* < .001 (medium effect size, Cohen’s *d* = .31), more often girls, *χ* ^2^(1) = 8.64, *p* = .003 (small effect, Cramer’s *V* < .01), and more often of a higher educational level, *χ* ^2^(4) = 33.55, *p* < .001 (small effect, Cramer’s *V* < .01). Only 20.3% of the non‐selected adolescents provided friendship quality data and 47.3% wrote a narrative, suggesting that non‐selection was mainly due to the absence of friendship quality data. This might have been the case because the questionnaire for friendship quality, the NRI, was at the end of the questionnaire, making that not all adolescents may have had time to fill out this questionnaire. For the adolescents who did provide relationship quality data, perceived friendship quality was higher for adolescents who were selected for our cross‐sectional sample than for the non‐selected adolescents, *t*(277.26) = −4.94, *p* < .001 (medium effect, Cohen’s *d* = .37).

Of all participants in the cross‐sectional sample, a limited number signed up for the longitudinal study. Of the full sample of Project‐Me (i.e., *N* = 1941), 10.8% wrote a narrative and had data on friendship quality in the second wave, and 10.1% in the third wave. As was the case for the cross‐sectional sample, missingness on perceived friendship quality, self‐event connections, and redemption across the three waves was found to be missing at random in the longitudinal sample, *χ* ^2^(83) = 85.49, *p* = .404. Compared to participants with only one wave of data on narratives and perceived friendship quality (i.e., our cross‐sectional sample), participants with at least two waves of data (i.e., longitudinal sample) were more likely to be female, *χ* ^2^(1) = 4.03, *p* = .045 (small effect size, Cramer’s *V* < .01), and of higher educational level, *χ* ^2^(4) = 43.51, *p* < .001 (small effect, Cramer’s *V* = .01). Moreover, participants with more than one wave of data were more likely to make a self‐event connection, *χ* ^2^(1) = 7.81, *p* = .005 (small effect, Cramer’s *V* = .01) at Wave 1, but there was no significant association with the presence of a redemption sequence, *χ* ^2^(1) = 1.48, *p* = .224.

#### Main analyses

**Figure 1:**
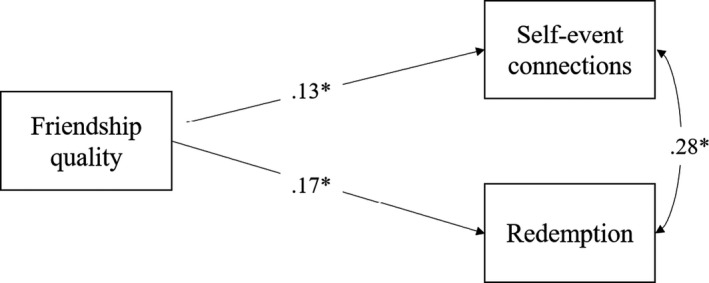
Path model detailing the cross‐sectional model with standardized regression estimates. *Note*. ^*^ *p* < .05

We examined the links of perceived friendship quality with self‐event connections and redemption sequences in two steps. In the first step, the cross‐sectional links were investigated. To do this, we used Wave 1 data. A cross‐sectional path model was first estimated, using perceived friendship quality as predictor and the categorical narrative identity constructs as the outcomes (see Figure 1). Age, gender, and education level were entered as control variables, as they have been found to be related to narrative identity characteristics and friendship quality (e.g., McLean, 2008).

**Figure 2:**
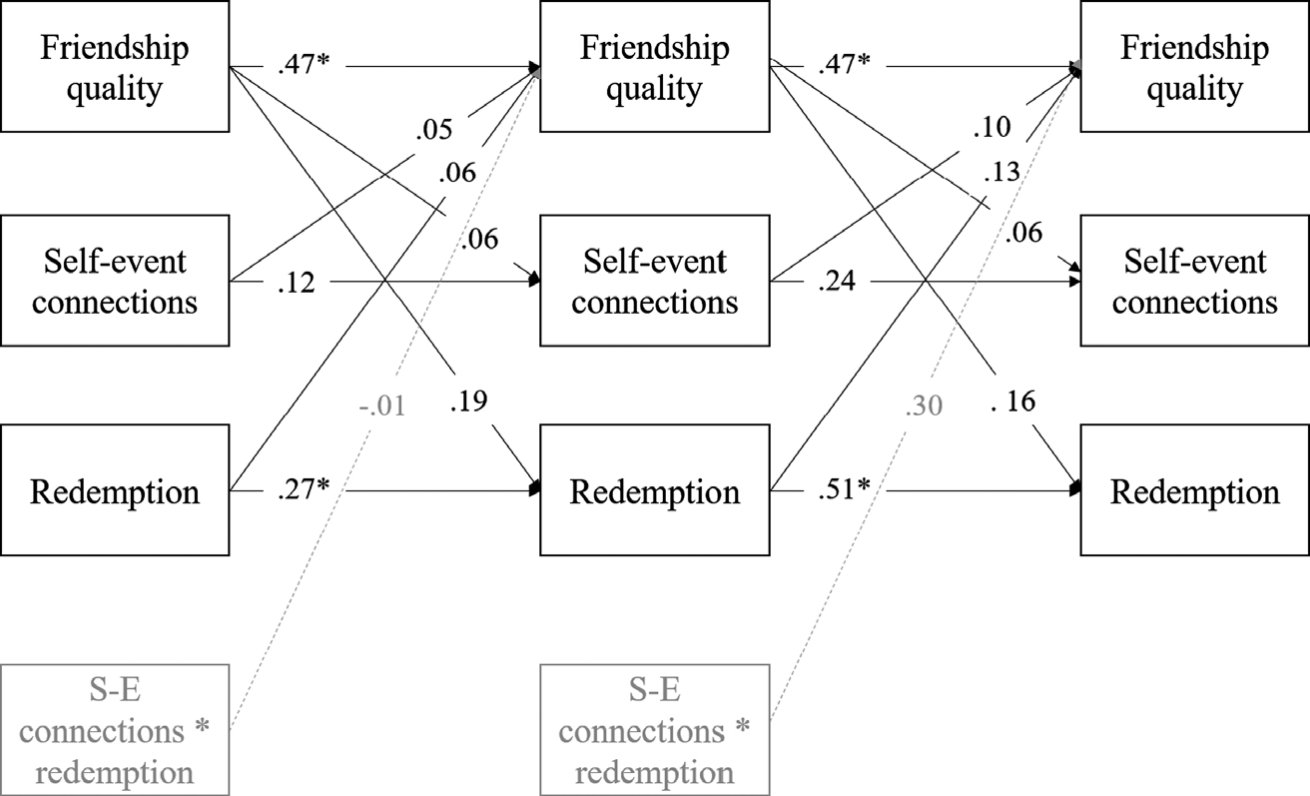
Path model detailing the longitudinal model with standardized regression estimates. *Note*. Only coefficients for longitudinal pathways are shown in the figure. All effects in the figure were estimated using the time‐invariant longitudinal model, with the exception of the interaction effects. These were estimated in the interaction model. ^*^ *p* < .05

In the second step, we examined how perceived friendship quality was related to the two narrative identity constructs over time in the longitudinal subsample. To do this, we estimated a path model with bidirectional associations of self‐event connections and redemption with friendship quality (see Figure 2; constructs and pathways in black). We further examined whether the model was time‐invariant by setting the paths Wave 1‐Wave 2 equal to those of Wave 2‐Wave 3. To reach convergence, covariances between the narrative identity characteristics were allowed to differ across waves. We then extended the model to include the interaction of self‐event connections and redemption as a predictor (see Figure 2; gray constructs and pathways).

All models were estimated in Mplus version 8.2 using a Bayesian estimator (Muthén and Muthén, (1998–2012)). As is the case for models using maximum likelihood estimation, Mplus uses full information from all observations for the Bayesian estimation of models. No model comparison indices are available yet in Mplus for models with categorical variables. Therefore, in Step 2 we made use of the Posterior Predictive P‐value (PPP) to compare model fit of the time‐invariant model to the time‐varying model, and of the interaction model compared to the final model (i.e., the time‐varying or time‐invariant model, depending on fit). The PPP is not a formal test of model comparison, but rather an indicator of good model fit, and its use can be compared to the χ^2^ *p*‐value. Similar to the χ^2^ *p*‐value, a PPP value of lower than .05 is indicative of poor model fit (Asparouhov et al., 2015). In cases of nested models with acceptable PPPs, it has been suggested that the most parsimonious model should be considered (Lubke & Muthén, 2007). Effects were tested for significance using a 95% credibility interval. Similar to a confidence interval, the interval indicates that there is a 95% probability that the true effect size falls into the interval. When the interval encompasses zero, effects were considered nonsignificant.

#### Robustness check

To test the robustness of our findings, we performed two sets of additional analyses. First, we reran our cross‐sectional model but with the direction of the regressions reversed. That is, with self‐event connections and redemption sequences as predictors of perceived friendship quality. Second, we reran our cross‐sectional and longitudinal analyses with an alternative definition of redemption. This coding uses stricter criteria for redemption. Specifically, it only includes narratives with negative‐affect state beginnings and positive‐affect state endings that also have an actual reinterpretation of the event taking place. This is slightly different from the definition we used, which is not dependent on the interpretation of the adolescent. To do this, we dichotomized the redemption scores into 0 (*no redemption; 0 or 1 in our original coding*) and 1 (*redemption; a 2 in our original coding*). The coding of redemption following this scheme is also presented in Table 2. Apart from the alternative coding of redemption, all other aspects of these analyses were similar to the main analyses. A summary of the results of the robustness analyses is reported at the end of the Results section; the results are reported more fully on pp. 3–6 of the Online Supplementary Material.

## RESULTS

Descriptive statistics for the cross‐sectional and longitudinal sample are reported in Table 1. Correlations between self‐event connections, redemption, and perceived friendship quality at Wave 1, Wave 2, and Wave 3 are provided in Table 3. Correlations were estimated for the cross‐sectional dataset (above the diagonal) and the longitudinal dataset (below the diagonal). At Wave 1, all correlations were positive as expected and significant. At later waves, the correlations were less strong, though overall consistent in direction.

**Table 3:** Correlations between the study variables in the sample

	1.	2.	3.	4.	5.	6.	7.	8.
1. Self‐event connections wave 1				.255*			.113*	
2. Self‐event connections wave 2	.136							
3. Self‐event connections wave 3	.131	.092						
4. Redemption wave 1	.325*	−.025	.151				.178*	
5. Redemption wave 2	.016	.116	−.151	.083				
6. Redemption wave 3	.148	.088	.289*	.145	.205			
7. Friendship quality wave 1	.107	.120	.061	.039	.245*	.130		
8. Friendship quality wave 2	.070	−.037	−.134	−.027	.273*	.038	.599*	
9. Friendship quality wave 3	.032	.060	.056	−.037	.267	.147	.472*	.685*

Note

Correlations above the diagonal are based on the cross‐sectional sample (*n* = 1087). Correlations under the diagonal were derived from the longitudinal sample (*n* = 186).

* *p* < .05.

### Linkages between perceived friendship quality and narrative identity

We first estimated the *cross‐sectional* links between perceived friendship quality, self‐event connections, and redemption (see Table 4). Perceived friendship quality was found to be related to a greater likelihood of making redemption sequences and of making self‐event connections. Thus, adolescents who reported higher friendship quality were more likely to make a redemption sequence and a self‐event connection in their turning point narrative at the same time point. In addition, having a higher educational level was related to making a self‐event connection. Finally, adolescents who made a self‐event connection were also more likely to make a redemption sequence. This suggests that adolescents who showed evidence of engaging in one kind of narrative identity processing, were also more likely to engage in other kinds.

**Table 4:** Estimates from the cross‐sectional model

	*b*	*Beta*	95% CI
Regression pathways			
Predictors of self‐event connections			
Age	0.06	0.04	[−0.03; 0.11]
Gender	0.07	0.04	[−0.06; 0.12]
Educational level	0.20*	0.19*	[0.11; 0.26]
Friendship quality	0.13*	0.13*	[0.06; 0.23]
Predictor of redemption			
Age	0.05	0.03	[−0.04; 0.10]
Gender	0.19	0.09	[−0.01; 0.18]
Educational level	0.06	0.06	[−0.01; 0.14]
Friendship quality	0.17*	0.17*	[0.08; 0.27]
Covariances			
Self‐event connections ↔ redemption	0.28*	0.28*	[0.18; 0.38]

Note

95% C.I. = 95% credibility interval based on the standardized estimate.

* *p* < .05.

Next, we estimated the *longitudinal* model with bidirectional links between perceived friendship quality, self‐event connections, and redemption (see Table 5). As both the time‐invariant model and the time‐varying model showed acceptable fit (PPP = .420 for the time‐invariant and PPP = .460 for the time‐varying model), findings from the time‐invariant model were interpreted (see Figure 2). The autoregressive coefficients were significant for perceived friendship quality and redemption. This suggests that adolescents who reported higher friendship quality and made redemption sequences at one wave tended to report higher friendship quality and tended to make redemption sequences at the next wave, respectively. For self‐event connections, whether or not adolescents made connections at one wave was not predictive of making self‐event connections one year later. Furthermore, perceived friendship quality did not significantly predict self‐event connections or redemption sequences, nor was the presence of these narrative characteristics predictive of perceived friendship quality at the next wave. Although not significant, effect sizes were quite similar to those found in the cross‐sectional model, especially for the link between friendship quality and redemption. Gender was also predictive of friendship quality, with girls reporting higher quality in friendships than boys did.

**Table 5:** Estimates from the longitudinal model

	*b*	*Beta*	95% C.I.	*b*	*Beta*	95% C.I.
	Wave 1 → Wave 2			Wave 2 → Wave 3		
Regression pathways						
Predictors of friendship quality						
Age	0.06	0.04	[−0.06; 0.14]	0.06	0.04	[−0.06; 0.13]
Gender	0.49*	0.24*	[0.12; 0.36]	0.49*	0.24*	[0.12; 0.35]
Educational level	< −0.01	< ‐ 0.01	[−0.10; 0.09]	< −0.01	< −0.01	[−0.10; 0.09]
Friendship quality	0.48*	0.47*	[0.35; 0.57]	0.48*	0.47*	[0.33; 0.61]
Self‐event connections	0.09	0.05	[−0.05; 0.14]	0.09	0.10	[−0.10; 0.28]
Redemption	0.11	0.06	[−0.03; 0.14]	0.11	0.13	[−0.06; 0.31]
Self‐event connections × Redemption	< −0.01	< −0.01	[−0.36; 0.32]	0.61	0.30	[−0.37; 0.92]
Predictors of self‐event connections						
Age	−0.13	−0.09	[−0.18; < −0.01]	−0.13	−0.08	[−0.17; < −0.01]
Gender	0.17	0.08	[−0.12; 0.29]	0.17	0.07	[−0.12; 0.28]
Educational level	0.09	0.06	[−0.11; 0.23]	0.09	0.06	[−0.11; 0.23]
Friendship quality	0.07	0.06	[−0.16; 0.27]	0.07	0.06	[−0.16; 0.27]
Self‐event connections	0.25	0.12	[−0.08; 0.30]	0.25	0.24	[−0.17; 0.55]
Predictors of redemption						
Age	−0.05	−0.03	[−0.13; 0.06]	−0.05	−0.03	[−0.11; 0.05]
Gender	0.33	0.14	[−0.05; 0.33]	0.33	0.12	[−0.04; 0.28]
Educational level	0.05	0.03	[−0.13; 0.19]	0.05	0.03	[−0.11; 0.16]
Friendship quality	0.22	0.19	[−0.03; 0.40]	0.22	0.16	[−0.03; 0.36]
Redemption	0.61*	0.27*	[0.08; 0.42]	0.61*	0.51*	[0.17; 0.71]
Correlated error terms at Wave 2 and Wave 3						
Friendship quality ↔ self‐event connections	−0.09	−0.13	[−0.38; 0.12]	0.16	0.23	[−0.07; 0.50]
Friendship quality ↔ redemption	0.13	0.19	[−0.08; 0.43]	−0.01	−0.01	[−0.39; 0.33]
Self‐event connections ↔ redemption	0.14	0.14	[−0.25; 0.49]	0.41	0.41	[−0.01; 0.75]

Note

All estimates were derived from the time‐invariant model, with the exception of the interaction term. 95% C.I. = 95% credibility interval based on the standardized estimate.

* *p* < .05.

We then added an interaction term to the model of the two types of narrative identity processes. This interaction term was added at Wave 1 as a predictor of perceived friendship quality at Wave 2, and at Wave 2 as a predictor of Wave 3. This resulted in poorer model fit (PPP = .220). Moreover, the interaction did not significantly predict friendship quality at Wave 2 or Wave 3. Following the suggestion of one of the reviewers, we also tested an interaction model with moderation of gender on the cross‐lagged paths of perceived friendship quality with self‐event connections and redemption, and vice versa. Like the paths in the time‐invariant longitudinal model, the four added interactions were set equal from Wave 1 to Wave 2 and from Wave 2 to Wave 3. This model showed poor fit to the data (PPP < .001) and was therefore not further interpreted.

Taken together, our cross‐sectional model indicated that quality of adolescents’ friendships is concurrently related to the presence of a redemption sequence and a self‐event connection in their turning point narrative. Moreover, the presence of a redemption sequence is also related to creating a self‐event connection. Longitudinally, we did not find support for a covariance between the presence of self‐connections and redemption sequences. Additionally, there was no significant effect of perceived friendship quality on narrative identity characteristics or vice versa, nor of the interaction term between the narrative identity characteristics on friendship quality.

### Robustness analyses

To test the robustness of our findings, we first estimated a cross‐sectional model in which the narrative identity characteristics were the predictors and perceived friendship quality was the outcome. Results from this model were largely the same as those in the original model, with both the making of a self‐event connection and a redemption sequence being related to higher friendship quality.

Next, we examined whether a different operationalization of redemption would alter our findings. To do this, we fitted the same models as before, with the only difference being the operationalization of the redemption variable. Findings regarding our variables of interest were highly similar for the cross‐sectional model. For the longitudinal model, they were mostly in agreement with the findings regarding our variables of interest of our main analyses, with the exception that there was now a significant covariance between self‐event connections and redemption at Wave 3. Similar to the main findings, the findings of the model including the interaction term evidenced no significant interaction effect. In sum, the robustness analyses and main analyses showed the same pattern of associations, in that there was a significant association between perceived friendship quality and narrative identity in the cross‐sectional model, but no significant predictive effects in either direction in the longitudinal model. The findings of these robustness checks are described more fully in the Online Supplementary Material, pp. 3–6, Tables S2–S4.

## DISCUSSION

The present study examined the association between perceived friendship quality in the friendship with the best friend and characteristics of adolescents’ narrative identities. Friendships of higher quality, that likely provide a safe basis and scaffolding, were expected to predict a more developed narrative identity, as indicated by a greater likelihood of creating a self‐connection and a redemption sequence in a turning point narrative (Fivush et al., 2006). In turn, we hypothesized that a more developed identity would contribute to higher friendship quality through better managing of social interaction goals and accomplishment of developmental tasks (Erikson, 1950; Swann, 2000). In addition, we postulated that especially redemption sequences for an event that was also linked to the self would be associated with higher friendship quality. Our findings provided support for cross‐sectional linkages between perceived friendship quality, self‐event connections, and redemption sequences. However, no significant longitudinal linkages were found.

### Association between perceived friendship quality and narrative identity

Cross‐sectionally, we found that perceived friendship quality was related to the presence of redemption sequences and self‐event connections. That is, adolescents who perceived their friendships as being of higher quality were better able to positively reinterpret negative events and link events to their self‐concept. This was true even after controlling for age, gender, educational level, and the presence of the other narrative identity characteristic. These findings indicate that two important developmental processes in adolescence, the creation of a well‐developed narrative identity and the formation of friendships of high quality, are linked (Bauer & McAdams, 2004; Erikson, 1950; McAdams et al., 2001).

Although we could not test for a statistical difference between the estimates, across our analyses the link between perceived friendship quality and self‐event connections appeared less robust than the link between friendship quality and redemption, as inferred from the smaller standardized coefficient estimate. This may reflect the difference in conceptualization of redemption sequences and self‐event connections. Specifically, whereas having a positive outlook on one’s experiences may almost always be a good thing—and may even reflect a broader positive interpretation style—linking experiences to the self may not always be (Klimstra & Denissen, 2017; McLean & Mansfield, 2011). Thus, the link with self‐event connections may be more diffuse, and as a result, less strongly associated with friendship quality. For instance, traumatic events such as sexual assault may result in connections of stability (i.e., “This happened to me because I’m a bad person”) or change (i.e., “I became much more weary of others because of this event”) that deteriorate adolescents’ friendships rather than contribute to them. More normative negative events such as an argument in a friendship or breaking up a friendship may also result in connections to the self that are not adaptive. Indeed, Banks and Salmon (2012) found that making negative self‐event connections, particularly in narratives concerning a low point in individuals’ lives, was related to lower well‐being, as measured by several dimensions of personal and social functioning such as self‐acceptance and positive associations with others. As such, it could be that a closer look at the content and valence of self‐event connections is needed to understand when making such connections is related to adaptive (highly qualitative), and when to maladaptive friendships.

Longitudinally, however, we found no significant links between perceived friendship quality and either self‐event connections or redemption. That is, higher friendship quality did not predict the presence of these narrative characteristics, or vice versa. As such, our findings provided only tentative evidence for either scaffolding theory (Fivush et al., 2006) or Erikson’s theory on developmental tasks (1950), as the cross‐sectional findings cannot tell us about directionality in the link between perceived friendship quality and narrative identity characteristics. Alternatively, the consistent cross‐sectional links suggest that a third variable may predict changes in both narrative identity and perceived friendship quality. For instance, people with certain personality traits such as low neuroticism or positive affectivity may be both more likely to make self‐event connections and redemption sequences, and experience their friendships as being of higher quality. Of course, it should be noted that while we found no support for theory proposing a predictive effect of relationship quality on narrative identity, in our study we only examined perceived quality of the friendship with the best friend. Other social contacts such as parents, siblings, or other friends may also help adolescents construct their narratives. In past work examining mothers and friends, these relationships have been found to be meaningful for narrative development (McLean & Jennings, 2012). Thus, more research using a variety of close relationships is needed to test scaffolding theory (Fivush et al., 2006) and Erikson’s theory (1950) more thoroughly.

It should be noted that due to a much smaller sample than for the cross‐sectional analyses, power for the longitudinal analyses was relatively low. In particular, while we had enough power to detect small effects (*f* ^2^ > .01) in the cross‐sectional sample, we could only detect small to medium effects (*f* ^2^ > .07) in the longitudinal sample. As such, it is possible that the reported cross‐sectional findings did not disappear, but rather were rendered nonsignificant due to a lack of sufficient power. Indeed, when examining the size of the estimated effects of the longitudinal model, we see that the estimates for the predictive effect of perceived friendship quality on narrative identity (i.e., effect sizes between 0.06 and 0.19; Table 5) are quite similar to the estimates of the cross‐sectional model (i.e., between 0.13 and 0.17; Table 4). Moreover, previous research using larger samples has often reported similar effect sizes for psychological constructs in longitudinal models (Adachi & Willoughby, 2015), suggesting that some of our longitudinal findings may indeed reflect existing effects that did not reach significance due to limited power. This may be especially true for the association between perceived friendship quality and redemption, which was more sizable than the association between perceived friendship quality and self‐event connections in the cross‐sectional model. Again, these findings were largely corroborated by the robustness analyses, which also evidenced effects in the longitudinal model that were similarly sized as the cross‐sectional effects, but were nonsignificant (see Online Supplementary Material, pp. 4–5, Tables S3 and S4).

Based on the similar effect sizes in the cross‐sectional and longitudinal models, our findings seem to tentatively suggest that perceived friendship quality may be important for the development of narrative identity, as operationalized as the creation of redemption sequences and self‐event connections, which would be in line with previous findings on scaffolding behavior in conversations with friends (McLean & Jennings, 2012; Pasupathi & Hoyt, 2009) and scaffolding theory (Fivush et al., 2006). Moreover, such links would provide a potential mechanism for previous findings of a predictive effect of friendship quality on identity (e.g., Meeus et al., 2002; De Moor et al., 2019). That is, it could be that more highly qualitative friendships help adolescents positively reinterpret negative events and make meaning of their experiences, thus contributing indirectly to adaptive identity development. However, more research using a larger sample is needed to examine whether these effects are indeed significant when power is higher, before such inferences can be made.

Yet, it should be noted that even if they had been significant, the size of these effects was rather small, suggesting that any association between perceived friendship quality and narrative identity is quite limited. As we examined the effect on narrative identity in one particular narrative, however, this may not be surprising. That is, if we were to examine the presence of self‐event connections and redemption in many different narratives, it is likely that how adolescents construct their narratives is less influenced by situational factors (e.g., the particular story, the setting in which they have to write it down) and more by their stage of narrative identity development. Indeed, previous work has shown that at a measurement occasion, there is less variation between individuals than between the narratives written by the same individual (McLean, Syed, & Shucard, 2016). Still, more longitudinal work using a larger sample of adolescents and narratives would be needed to test this possibility. Another potential explanation for the modest effect sizes is that perceived friendship quality as a construct might be too broad to capture the scaffolding behavior that has been theorized to be the driving mechanism behind the influence of high‐quality friendships on narrative identity (Fivush et al., 2006). Relationships that are perceived as being of high quality can contain both promotive (e.g., scaffolding) and hampering (e.g., co‐rumination) behaviors, which may not all be related to a more developed identity (Rose, 2002). Therefore, examining more specific interaction behaviors may evidence stronger associations with the presence of self‐event connections and redemption sequences, as has been the case in conversational research contexts (McLean & Jennings, 2012; Pasupathi & Hoyt, 2009).

Finally, although not the main focus of our study, our analyses resulted in a noteworthy finding with regard to stability. Our longitudinal analyses evidenced moderate and small to moderate stability for perceived friendship quality and redemption, respectively, but low stability for self‐event connections (i.e., effect sizes of 0.12 and 0.24). The low stability estimate could not be explained by a drift in the coding of self‐event connections, as a subsample of 30 narratives from Wave 1 were recoded after the completion of the coding for Wave 3, which resulted in a reliability estimate of (κ) .92 with the initial codes. However, it is possible that making self‐event connections is more situation‐dependent than redemption, which may be reflective of having a more positive outlook on life in general. In contrast, whether or not an adolescent makes a self‐event connection may not only be dependent on an underlying disposition, but also on the event they write about in their turning point narrative. Given that the characteristics of narrative identity were assessed in a singular event, it may therefore not be surprising that the presence of self‐event connections was somewhat unstable.

### Limitations

The current study examined the association between perceived friendship quality and two characteristics of narrative identity, using narrative data from over a thousand adolescents who provided in‐depth narratives on a turning point moment in their lives. In the present study, we combined this narrative data with questionnaire data on social relationships, resulting in a multi‐method design, which limits shared‐method bias (Podsakoff et al., 2012) and thus provides more robust findings.

However, some limitations of the present study need also be mentioned. First, while the aim of the study was to examine inter‐individual differences in narrative identity, it would also be interesting to examine how identity and perceived friendship quality are associated with each other within‐person. That is, while we can now conclude that adolescents who have higher quality friendships are more likely to make self‐event connections and redemption sequences, a within‐person approach would provide insight in the process through which adolescents develop their identity. That is, it could indicate whether friendship processes are actually a mechanism through which adolescents develop their own identity, thus helping us better understand the process of narrative identity construction. Future studies with larger samples should therefore aim to disentangle between‐person from within‐person variance, for instance by using an intensive individual‐focused mixed‐method approach.

Second, given gender differences in friendship quality (e.g., De Goede et al., 2009) and following the suggestion from one of the reviewers, we examined moderation effects of gender on the cross‐lagged associations of friendship quality and narrative identity in the longitudinal model. This model showed poorer fit than the model without gender moderation, perhaps in part due to its relative complexity and the relatively small sample in which it was examined. As such, findings from this model were not interpreted. However, future work with larger samples should study gender differences in the association between friendship quality and narrative identity in greater detail. Such studies may also consider using a more inclusive definition of gender, for instance by including a non‐binary option or by capturing gender as a continuous rather than a categorical variable. It is likely that a nuanced conception of gender will more accurately reflect actual differences in the population.

Third, and related to the previous points is the issue of power in the longitudinal analyses. Specifically, less than 10% of the participants of the total cross‐sectional sample also consented to participate in the longitudinal study, thus providing information on narrative identity and the quality of their friendships at Wave 2 and/or 3. The much smaller size of the longitudinal sample was likely because of the fact that active parental informed consent was required for the 2^nd^ and 3^rd^ wave. The IRB approved a procedure in which adolescents had to bring a letter home to their parents, after which their parents had to mail back the signed consent letter. This procedure likely did not facilitate participation. Moreover, missing data in either of the measures also contributed to the reduced sample size. In particular, as the NRI was near the end of the questionnaire, some adolescents did not get to fill out the questions on friendship quality (38.9% at Wave 1). Adolescents who participated in more than one wave had more developed identities, as evidenced by a greater likelihood of making a self‐event connection. This suggests that in addition to a smaller sample, the longitudinal sample may also reflect a subgroup of individuals who are generally better‐adjusted (Bauer & McAdams, 2004; Erikson, 1950, 1968). This may have resulted in an underestimation of the association between perceived friendship quality and narrative identity characteristics. In future studies, it is therefore important to not only use larger samples, but also samples that cover a wider range of adolescents.

Fourth, in the present study we examined the association of friendship quality and narrative identity. This link was substantiated by evidence that a high‐quality friendship may form a context in which adolescents are listened to and are stimulated to explore and understand their experiences (e.g., McLean & Jennings, 2012; Pasupathi & Hoyt, 2009), as well as a safe basis from which adolescents can explore and develop their identity beyond the immediate friendship context (Ainsworth, 1989; Bowlby, 1969; Furman & Buhrmester, 2009). Yet, adolescents may differ in the types of friendships they have and they may even have different friendships of different types. Importantly, not every high‐quality friendship necessarily entails adolescents discussing self‐relevant topics. For instance, some friendships may also be based on shared interests or activities. Although such friendships may not affect identity in a direct manner (i.e., through discussion of important experiences), we would expect that such friendships still provide a safe basis for identity exploration more indirectly (e.g., having a good friend may give adolescents the confidence to explore more extensively what they are looking for in a romantic relationship). It is important that future research examines whether the association of friendship quality with narrative identity is indeed partially mediated by the topics that adolescents discuss with their friends, as was presumed in the present study.

Finally, reliability of the coding of redemption sequences (κ = .63 for the cross‐sectional sample and .66 for the longitudinal sample) was relatively low, even though it was still acceptable (see recommendations by Syed & Nelson, 2015). In contrast, several recent studies that used the same coding system (e.g., Alea, 2018; Bauer et al., 2019; McCoy & Dunlop, 2017) all reported higher reliability. With the exception that these studies often focused on adult samples, there were no clear differences in study design that may explain the discrepancy in reliability between this and previous work. Reliability may attenuate effect size (Kanyongo et al., 2007) and as such it could be that the (absence of) findings of the study are in part confounded by the relatively low reliability of the coding of redemption.

## CONCLUSION

The present study examined cross‐sectional and longitudinal links between the perceived quality of adolescents’ best friendships and autobiographical narratives. Taken together, our findings showed that adolescents who experience higher friendship quality in the relationship with their best friend were indeed more likely to make self‐event connections and redemption sequences in their turning point narratives. However, the linkages between perceived friendship quality and narrative identity characteristics were not significant in the longitudinal analyses, despite showing similar sized estimates to the cross‐sectional analyses. Based on the present cross‐sectional and longitudinal findings, we believe it might be worthwhile to examine whether the degree of friendship quality adolescents experience does predict adaptive relative changes in narrative identity features, and vice versa, in a future larger sample.

## Supporting information

Table S1‐S5
