# Supplementary material for: With a little help from my friends? Perceived friendship quality and narrative identity in adolescence
Source: J Res Adolesc. 2021 Feb 23;31(2):384–401. doi: 10.1111/jora.12605 (PMC8248078; doi:10.1111/jora.12605)
Supplement: Supplementary file 1 — Table S1‐S5 [file JORA-31-384-s001.docx]

**Supplementary Material**

Table S1. Deviations From the Pre-Registered Plan.

| Initial plan | Deviation | Rationale |
| --- | --- | --- |
| Examine the cross-sectional link of perceived friendship quality with self-event connections and redemption in a sample of 509 adolescents. | Examined the cross-sectional link of perceived friendship quality with self-event connections and redemption in a sample of 1,087 adolescents. | Sample size was incorrectly estimated for the pre-registration. However, the inclusion criteria did not change. |
| Examine links with self-event connections and redemption separately for received support in the friendship with the best friend and provided support. | Examined the link with self-event connections and redemption of a combined measure of received and provided support, which we named perceived friendship quality. | The NRI received and provided support subscales demonstrated high correlations subscales (*r*_range_ = .86-.91), which impacted the estimation of our models. |
| Examine a cross-sectional model in which self-event connections and redemption predict perceived friendship quality, and the reversed model in the Supplementary Material as robustness check. | Examined a cross-sectional model in which perceived friendship quality predicts self-event connections and redemption, and the reversed model in the Supplementary Material as robustness check. | We felt that a model with perceived friendship quality better mapped onto our theoretical background and research questions (i.e., whether individual differences in narrative identity could be predicted by differences in friendship quality). |
| Use a definition of redemption that includes only narratives in which an explicit reinterpretation by the participant was made, in addition to a switch from an initial negative affect state to a positive affect end state. As a robustness check, we rerun our models using a less strict definition of redemption in which there does not need to be a reinterpretation. | Used the less strict definition of redemption in our main analyses. Models with the stricter definition of redemption are described in the Supplementary Material as robustness check. | The less strict definition of redemption is more in line with previous work on redemption (e.g., McLean & Breen, 2009). |
| Due to the dichotomous nature of our narrative identity measures, use the WLSMV estimator in Mplus. | Used the Bayesian estimator in Mplus. | We encountered problems with the weight matrix in the interaction model that could not be alleviated using WLSMV estimation. As such, we opted to use a Bayesian estimator, which does not rely on a weight matrix. |

**Robustness Checks
 Findings from the reversed cross-sectional model.** We tested a cross-sectional model with reversed direction of effects. That is, we estimated a model in which self-event connections and redemption sequences were the predictors and perceived friendship quality was the outcome. Findings from these analyses showed that as in our original model, redemption and self-event connections were positively related to friendship quality. Gender was also related to perceived friendship quality, with girls reporting higher friendship quality compared to boys. Finally, and similar to the original model, self-event connections and redemption sequences were significantly related.

Table S2. Estimates From the Reversed Direction Cross-Sectional Model.

|  | *b* | *Beta* | 95% C.I. |
| --- | --- | --- | --- |
| **Regression pathways** |  |  |  |
| Predictors of friendship quality |  |  |  |
| Age | 0.06 | 0.04 | [-0.01; 0.09] |
| Gender | 1.07* | 0.52* | [0.48; 0.56] |
| Educational level | -0.07 | -0.06 | [-0.11; -0.01] |
| Self-event connections | 0.13* | 0.06* | [0.01; 0.12] |
| Redemption | 0.19* | 0.09* | [0.03; 0.13] |
| **Covariances** |  |  |  |
| Self-event connections ↔ redemption | 0.05* | 0.18* | [0.13; 0.24] |

*Note*. 95% C.I. = 95% credibility interval based on the standardized estimate.
* *p* < .05

**Findings from the analyses using the alternative redemption measure.** As in the cross-sectional model with our original redemption variable, educational level and friendship quality were related to making a self-event (see Table S3). With regards to redemption, we also found that having higher friendship quality predicted making a redemption sequence, as was the case in the general model. In addition, we similarly found a significant covariance between self-event connections and redemption.
 Similar as for the longitudinal model using the original redemption variable, we found significant autoregressive coefficients for friendship quality and redemption (see Table S4). The sizes of the estimates in this model were similar to those of the original model, and to those of the cross-sectional model. As in the original model, girls reported higher friendship quality than boys. In addition, older adolescents were less likely to make self-event connections. Finally, we found a significant covariance between self-event connections and redemption, but only at Wave 3.

Table S3. Estimates From the Cross-Sectional Model With the Alternative Redemption Variable.

|  | *b* | *Beta* | 95% C.I. |
| --- | --- | --- | --- |
| **Regression pathways** |  |  |  |
| Predictors of self-event connections |  |  |  |
| Age | 0.06 | 0.04 | [-0.03; 0.11] |
| Gender | 0.07 | 0.03 | [-0.06; 0.12] |
| Educational level | 0.20* | 0.18* | [0.11; 0.26] |
| Friendship quality | 0.13* | 0.13* | [0.06; 0.22] |
| Predictors of redemption |  |  |  |
| Age | 0.01 | < 0.01 | [-0.07; 0.08] |
| Gender | 0.07 | 0.03 | [-0.06; 0.14] |
| Educational level | 0.08 | 0.07 | [-0.01; 0.17] |
| Friendship quality | 0.25* | 0.25* | [0.15; 0.36] |
| **Covariances** |  |  |  |
| Self-event connections ↔ redemption | 0.43* | 0.43* | [0.33; 0.53] |

*Note*. 95% C.I. = 95% credibility interval based on the standardized estimate.
* *p* < .05

Table S4. Estimates From the Longitudinal Model.

|  | *b* | *Beta* | 95% C.I. | *b* | *Beta* | 95% C.I. |
| --- | --- | --- | --- | --- | --- | --- |
|  | **Wave 1 → Wave 2** | | | **Wave 2 → Wave 3** | | |
| **Regression pathways** |  |  |  |  |  |  |
| Predictors of friendship quality |  |  |  |  |  |  |
| Age | 0.05 | 0.03 | [-0.06; 0.13] | 0.05 | 0.03 | [-0.06; 0.13] |
| Gender | 0.49* | 0.25* | [0.13; 0.37] | 0.49* | 0.25* | [0.13; 0.36] |
| Educational level | < 0.01 | < 0.01 | [-0.09; 0.10] | < 0.01 | < 0.01 | [-0.09; 0.10] |
| Friendship quality | 0.49* | 0.48* | [0.36; 0.58] | 0.49* | 0.49* | [0.34; 0.63] |
| Self-event connections | 0.10 | 0.05 | [-0.04; 0.15] | 0.10 | 0.11 | [-0.09; 0.31] |
| Redemption | 0.03 | 0.01 | [-0.07; 0.10] | 0.03 | 0.03 | [-0.18; 0.23] |
| Interaction term | -0.11 | -0.05 | [-0.43; 0.27] | 0.65 | 0.32 | [-0.50; 0.94] |
| Predictors of self-event connections |  |  |  |  |  |  |
| Age | -0.10 | -0.07 | [-0.16; 0.04] | -0.10 | -0.06 | [-0.16; 0.03] |
| Gender | 0.17 | 0.08 | [-0.13; 0.29] | 0.17 | 0.08 | [-0.12; 0.29] |
| Educational level | 0.08 | 0.06 | [-0.12; 0.23] | 0.08 | 0.06 | [-0.12; 0.22] |
| Friendship quality | 0.06 | 0.05 | [-0.17; 0.27] | 0.06 | 0.05 | [-0.17; 0.27] |
| Self-event connections | 0.22 | 0.11 | [-0.10; 0.28] | 0.22 | 0.22 | [-0.21; 0.53] |
| Predictors of redemption |  |  |  |  |  |  |
| Age | -0.06 | -0.04 | [-0.11; 0.05] | -0.06 | -0.03 | [-0.09; 0.04] |
| Gender | 0.46 | 0.20 | [-0.01; 0.40] | 0.46 | 0.16 | [-0.01; 0.33] |
| Educational level | 0.04 | 0.02 | [-0.13; 0.19] | 0.04 | 0.02 | [-0.11; 0.16] |
| Friendship quality | 0.18 | 0.15 | [-0.06; 0.37] | 0.18 | 0.13 | [-0.05; 0.32] |
| Redemption | 0.65* | 0.26* | [0.11; 0.40] | 0.65* | 0.53* | [0.25; 0.71] |
| **Correlated error terms at Wave 2 and Wave 3** | |  |  |  |  |  |
| Friendship quality ↔ self-event connections | -0.10 | -0.14 | [-0.39; 0.12] | 0.16 | 0.24 | [-0.08; 0.50] |
| Friendship quality ↔ redemption | 0.14 | 0.20 | [-0.11; 0.46] | 0.02 | 0.02 | [-0.35; 0.36] |
| Self-event connections ↔ redemption | 0.19 | 0.19 | [-0.20; 0.54] | 0.67* | 0.67* | [0.27; 0.93] |

*Note*. All estimates were derived from the time-invariant model, with the exception of the interaction term.
95% C.I. = 95% credibility interval based on the standardized estimate.
* *p* < .05

Table S5. Overview of the Items of the Perceived Friendship Quality Scale.

| English | Dutch |
| --- | --- |
| How much do you seek out your best friend when you’re upset? | Hoe vaak ga je naar je beste vriend(in) als je van streek bent? |
| How much does your best friend encourage you to try new things that you’d like to do but are nervous about? | Hoe vaak moedigt je beste vriend(in) je aan nieuwe dingen te doen die je wel zou willen maar niet goed durft, of waar je tegenop ziet? |
| How much does your best friend turn to you for comfort and support when s/he is troubled about something? | Hoe vaak zoekt je beste vriend(in) jou op voor troost en hulp als hij of zij ergens mee zit? |
| How much do you encourage your best friend to try new things that s/he would like to do but is nervous about? | Hoe vaak moedig jij je beste vriend(in) aan nieuwe dingen te doen die hij of zij wel zou willen maar niet goed durft, of waar hij/zij tegenop ziet? |
| How much do you turn to your best friend for comfort and support when you are troubled about something? | Hoe vaak zoek je je beste vriend(in) op voor troost en steun als je ergens mee zit? |
| How much does your best friend encourage you to pursue goals and future plans? | Hoe vaak moedigt je beste vriend(in) je aan om je plannen en dromen voor de toekomst na te streven? |
| How much does your best friend turn to you when s/he is worried about something? | Hoe vaak komt je beste vriend(in) bij jou als hij of zij zich ergens zorgen over maakt? |
| How much do you encourage your best friend to pursue his/her goals and future plans? | Hoe vaak moedig jij je beste vriend(in) aan om zijn of haar plannen en dromen voor de toekomst na te streven? |
| How much do you turn to your best friend when you’re worried about something? | Hoe vaak ga je naar je beste vriend(in) als je je ergens zorgen over maakt? |
| How much does your best friend show support for your activities? | Hoe vaak steunt je beste vriend(in) je bij de dingen die je doet? |
| How much does your best friend seek you out when s/he is upset? | Hoe vaak komt je beste vriend(in) bij jou als hij of zij van streek is? |
| How much do you show support for your best friend’s activities? | Hoe vaak steun jij je beste vriend(in) bij de dingen die hij of zij doet? |

*Note.* The participants responded to the Dutch version of the items.
